# Mind and Spirit. Chaplaincy and Spiritual Care in Inpatient Psychiatry – a Qualitative Study

**DOI:** 10.1192/bjo.2022.187

**Published:** 2022-06-20

**Authors:** Elias Diamantis, Rupert Miles-Marsh, Anita Stowell

**Affiliations:** Nottinghamshire Healthcare NHS Foundation Trust, Nottingham, United Kingdom

## Abstract

**Aims:**

Introduction. Despite society's secularisation, as of 2019 only 38.4% of the population of England and Wales identified as “No Religion”. The integration of chaplaincy and spiritual care teams into health services varies widely and we undertook this qualitative research to better understand the spiritual needs on psychiatric wards.

**Methods:**

Between October 2021 and January 2022, we carried out semi-structured interviews with 10 patients and 10 staff-members, convenience sampled from acute General Adult Wards. The interviews were approximately 10–15 minutes long, documented in shorthand, compiled, and analysed thematically.

**Results:**

**Themes (P = patient, S = staff member)**

**1. Religion and belief, or lack of it, defies categorisation**

P1 (36M) identified as Christian but didn't really believe, whilst S2 (Nurse Clinical Team Leader) professed no religion but prayed that her sister would be healed. P7 (59F) was brought up Christian but thought religion was a fantasy. P2(21M) identified as Wiccan but thought all religions hold truth.

**2. An incarnational, embodied service**

P9 (33F) wished chaplains wandered around the wards and S10 (F1 Junior Doctor) praised their presence in general hospitals. P1 wanted a “prayer circle” and S5 (Student Nurse) suggested weekly worship services.

**3. Space to “be”**

S10 liked an empty chapel to think in and P4 (29M) said he was Lacking space for reflection and meditation.

**4. Unmet needs**

P9 felt abandoned by God during the admission and her vicar had recently died. She wanted someone to sit, pray with her and point her to helpful scriptures but was not aware of the existence of chaplaincy. Of the patients, only P3 knew how to contact the service and S8 said it was rarely discussed by the MDT.

**5. Caution, ignorance and suspicion**

S1 and S8 said chaplaincy visits are sometimes distressing for patients preoccupied with devils and demons and P5 (26M) was worried they'd judge him.

**6. Links with wider faith communities**

P6 (46F) would like to attend church with her family, P4(29M) would like to know where he could go to worship and S2 was also curious of what's available outside hospital.

**Conclusion:**

**Discussion and clinical implications**

Despite limitations of small size and recruitment bias, the themes emphasise the complexity of understanding someone's spirituality. It highlights a call for a more visible presence and thoughtful consideration of what a spiritual need is and how it can be met.

Ward visits should be prioritised, having recently been limited by COVID-19 restrictions. Patient information and staff education regarding chaplaincy and spiritual care is urgently needed on psychiatric inpatient wards.

